# Myocardial Infarction as a Consequence of Mitochondrial Dysfunction

**DOI:** 10.2174/1573403X19666230508114311

**Published:** 2023-10-02

**Authors:** Pranay Wal, Namra Aziz, Yash Kumar Singh, Ankita Wal, Sourabh Kosey, Awani Kumar Rai

**Affiliations:** 1 PSIT-Pranveer Singh Institute of Technology (Pharmacy), Bhauti, Kanpur, UP-209305, India;; 2 Department of Pharmacy Practice, NIMS Institute of Pharmacy, NIMS University, Jaipur, Rajasthan, India

**Keywords:** Myocardial Infarction (MI), mitochondrial dysfunction, calcium overload, reactive oxygen species, cell damage, oxidative stress apoptosis

## Abstract

Acute myocardial infarction is an event of myocardial necrosis caused by unstable ischemic syndrome. Myocardial infarction (MI) occurs when blood stops flowing to the cardiac tissue or myocardium and the heart muscle gets damaged due to poor perfusion and reduced oxygen supply. Mitochondria can serve as the arbiter of cell fate in response to stress. Oxidative metabolism is the function of mitochondria within the cell. Cardiac cells being highly oxidative tissue generates about 90% of their energy through oxidative metabolism. In this review, we focused on the role of mitochondria in energy generation in myocytes as well as its consequences on heart cells causing cell damage. The role of mitochondrial dysfunction due to oxidative stress, production of reactive oxygen species, and anaerobic production of lactate as a failure of oxidative metabolism are also discussed

## 1 INTRODUCTION

Coronary heart diseases are the leading cause of morbidity throughout the world [1]. Around 17.5 million deaths were reported worldwide in 2012 (31% of the total deaths) [2] and are expected to increase up to 24.2 million by 2030 [3]. Myocardial infarction is a chronic heart disease that arises due to plaque formation within the coronary artery. Severe deposition of cholesterol and fat within the blood vessels occurs and gives rise to a condition known as ischemia. It is widely acknowledged that ischemia and reperfusion lead to mitochondrial, as well as cellular, damage in cardiac cells [4]. Besides this, failure of mitochondria or mitochondrial dysfunction also plays a significant role in the progression of myocardial infarction [5].

Myocardial infarction (MI) is a term used to describe the pathological condition of the heart which is characterized by the reduced blood supply to the cardiac tissue due to ischemia in the coronary artery. This cardiac ischemia causes damage to cardiac tissue due to reduced oxygen supply, thus heart attack dominates. Myocardial infarction can also be a progressive result of angina pectoris [6].

The usual mechanisms of myocardial infarction include rapture or erosion of lipid-laden atherosclerotic plaque in the coronary artery [7, 8]. As plaque formation is a gradual process, however, is more severe when it occupies 75% of the space of blood vessels supplying to the heart. Further restriction in blood flow increases the oxygen supply and the onset of angina occurs [9]. Increased troponin levels can also be observed in diseases other than myocardial infarction such as myocarditis, cardiac injury, renal, cardiac, and respiratory failure as well as septic shock (Table **1**) [10].

### 1.1 Risk Factors

Diet, fat, cholesterol, salt, smoking, nicotine, alcohol, and drugs certain other pathological conditions such as obesity, chronic kidney diseases, diabetes, and other parameters such as old age, and lack of physical activity in women atherosclerotic coronary disease. It is also demonstrated that several risk factors usually associated with coronary disease and atherothrombotic type 1 myocardial infarction, including hyperlipidemia, diabetes mellitus, and a prior history of coronary artery disease, are also important predictors of future type 2 myocardial infarction [11-14].

### 1.2 Types

Acute myocardial infarction can be categorized on the basis of the presence or absence of ST-segment when observed on ECG [15]. Further classified into six: TYPE 1(infarction due to coronary antherothrombosis), TYPE2 (infarction due to imbalance between demand and supply), TYPE3 (infarction causing sudden death without biomarker in ECG), TYPE4a (infarction which results from coronary intervention), TYPE4b (infarction related thrombosis), TYPE5 (infarction related to coronary artery bypass grafting) [16, 17].

## 2 THE PATHOLOGICAL CONSEQUENCES OF MYOCARDIAL ISCHEMIA

Ischemia in the heart is a result of the disruption of atherosclerotic plaque [15,18]. Angina pectoris for a prolonged period and its precipitation also give rise to condition ischemia [18]. In humans, the cells of the heart also known as cardiomyocytes have the tendency to reserve phosphate as an energy source to maintain contractility for a period of total ischemia. Besides the availability of high energy sources, several pathways contribute to induced almost immediate functional depression [19, 20]. The generation of inorganic phosphate originating from creatine phosphate reserve inhibits the function of the contractile protein [21].

At the early stage of the cardiac ischemic condition, the heart is fully reversible if the flow of blood gets restored within 4-5 minutes after coronary occlusion. Meanwhile, a longer duration of the ischemic condition by functional depression despite full restoration of flow can cause cell death, even if the duration of coronary occlusion is not long enough to cause cardiac cell death [22].

Generation and accumulation of reactive oxygen species (ROS), as well as some amount of organophosphate in the early stage of reperfusion, may lead to the oxidation of contractile protein which delays the response and functional recovery [23].

Disturbance in the homeostasis of calcium in cardiomyocytes also plays a role in mediating dysfunction in the myocardium [24, 25]. A specific mechanism is involved in systolic dysfunction which may also be due to calcium overload, induced protease activation, and decreased responsiveness of sarcomere protein to calcium. Reactive oxygen species (ROS) are also known to play a role in the alteration of calcium homeostasis as a free radical [26].

### 2.1 Diastole Dysfunction

**Fig. (1):**
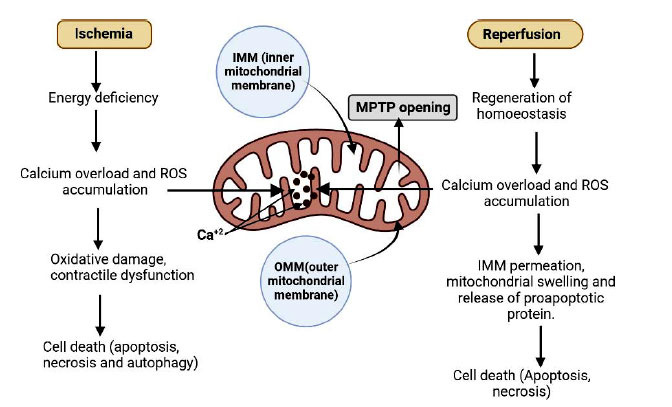
Diagrammatic representation of mitochondrial-induced ischemia and reperfusion. IMM (inner mitochondrial membrane), MPTP (mitochondrial permeability transition pore), and OMM (outer mitochondrial membrane). ROS (reactive oxygen species).

In the account of systole dysfunction, myocardial dysfunction may reduce contractility of the ventricle causing diastolic dysfunction. It may be either due to the overproduction of metabolic products such as lactate or due to hyperosmolar conditions that cause an increase in interstitial water and a reduction in myocardial compliance [27]. Although an imbalance between energy production and utilization may cause impaired relaxation and diastolic filling (Fig. **1**) [28, 29].

## 3 ROLE OF MITOCHONDRIA IN CARDIAC ISCHEMIA AND REPERFUSION

Ischemia causes cessation of aerobic and impacts anaerobic glycolysis which causes energy deficiency and imbalance in homeostasis. This phenomenon results in cytosolic and mitochondrial accumulation of ROS, calcium overload, and recurrent acidosis [30, 31]. Ischemia causes myocardial cell death by means of apoptosis and necrosis. Reperfusion causes rapid cellular regenerization and also corrects the intracellular pH by the removal of metabolites [32]. Rapid reperfusion also restores calcium overloading by establishing cellular homeostasis. Increased levels of mitochondrial calcium and ROS agonize MPTP opening which causes permeation of IMM and OMM and leads to cell death [33, 34].

The mitochondrial permeability transition pore (MPTP) is a pore that is present on the inner mitochondrial membrane (IMM) that remains closed under normal physiological conditions. At the time of certain stress stimuli, MPTP opens to form a high conductance pore that permeates the IMM to permit the equilibrium of solute of 1.5kDa [35]. Further MPTP opening leads to swelling of mitochondria and dissipates the membrane gradient thus inhibiting the ox-phos [36]. Rapture of the outer mitochondrial membrane occurs due to excess swelling and release of apoptotic protein such as cytochrome C occurs [36]. Based on the intensity of MPTP opening and the number of mitochondria affected damage or even cell death occur by means of apoptosis and necrosis. MPTP openings are directly involved in many pathological conditions [37]. Activators and inhibitors of MPTP opening primarily include activator-high calcium level, ROS, and inorganic phosphate and inhibition by low pH [38, 39]. Acidosis contributes to antagonizing MPTP opening, while inhibition of opening prevents the escape of ROS from the mitochondrial matrix to cytosol, thus, increasing the overall ROS accumulation [40].

### 3.1 A Metabolic Consequence of Ischemia and Reperfusion

Energy as a fuel is required by the cardiomyocyte for proper contractility of the myocardium. Mitochondrial, being the powerhouse of the cell, is the membrane-bound organelles responsible for energy production [41, 42]. The anaerobic process of glycolysis formation becomes the primary source of energy production which results in the accumulation of lactate in myocardium ischemia [43]. As mentioned above, the heart is a highly oxidative tissue though most of the time it undergoes aerobic metabolism for energy generation [44, 45]. During ischemia, the oxygen supply reduces, and the non-esterified fatty level increases which is a result of lipid breakdown rather than cessation of fatty acid oxidation [46, 47]. Under this condition, the tricarboxylic acid cycle (TCA) gets completely blocked thereby no energy is available by oxidative phosphorylation which ultimately leads to the accumulation of NADH whereby the ATP level is still maintained by glycolysis [46]. ATP generated by the glycolysis pathway contributes to less than 5% of the total ATP consumed by the heart of an adult [48]. Ischemia is a result of a large accumulation of lactate and a decrease in cytoplasmic PH and glycolysis is also inhibited [27]. ATP levels fall slowly which reduces to 30-40% of ATP with 30 minutes of ischemia. However, this changes markedly with NADH increasing sharply [49, 50]. Ionic concentration in sarcoplasm also changes rapidly in response to low ATP [51]. Over the longer duration of ischemia, it also suppresses the DNA and protein synthesis. On reperfusion electron transfer and ATP synthesis start and internal cytoplasmic PH is restored to 7 [52].

**Fig. (2):**
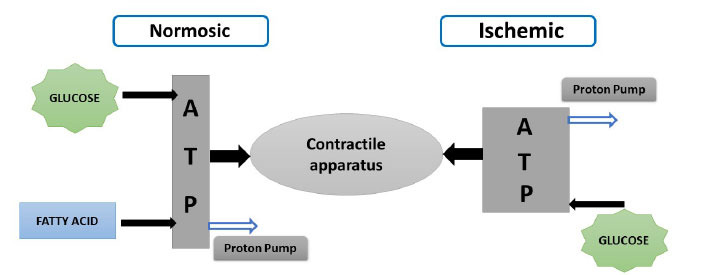
ATP balance in heart mitochondria.

**Fig. (3):**
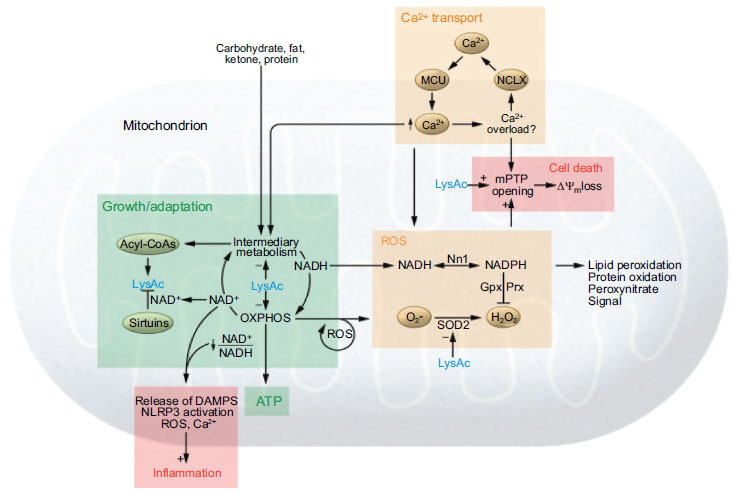
Maladaptive mechanism connecting mitochondrial dysfunction and progression of heart failure.

Ischemia as a result of atherosclerosis or plaque formation within the coronary artery may result in sudden cessation of oxidative phosphorylation thus cardiac myocyte uses another pathway for ATP production. Creatine phosphate as high-energy source gets exhausted rapidly [53]. At the stage of poor perfusion glucose supply to the myocyte reduces which is a main substrate for glycolysis in the ischemic heart. Thus, the anaerobic process of energy production cannot replace the more efficient energy ATP-producing capacity of oxidative phosphorylation, therefore, as a result, ATP gets consumed faster and faster as it is produced [54-57]. Intracellular accumulation of lactic acid by anaerobic process leads to acidosis that inhibits several enzymes of the glycolytic pathway [58]. ATP level in ischemic conditions is associated with the development of irreversible cardiomyocyte changes (Figs. **2** and **3**).

### 3.2 Normal Cardiomyocyte Cellular Function and Metabolism

Fatty acid is the primary source of energy for cardiac cell function. Approximately 60-70% of its energy requirement is fulfilled by this fatty acid. Approximately 25-30% of the space of cardiac cells is occupied by mitochondria [59]. The two types of mitochondria can be seen in a cardiac cell on the basis of position and extraction ‘interfibrilier’ and ‘subsacrolemmel’. Mitochondria also pass the calcium transport system with several enzymes in the oxidative metabolism pathway, thus calcium also serves as a secondary messenger in contractility as well as mitochondrial metabolism [60]. Energy balance in cardiac cells is maintained by the oxidation of glucose and lactate [61]. The heart is a highly oxidative tissue and about 90% of its total energy is a result of aerobic metabolism. Even with the abundant availability of oxygen in cardiac tissue, some of the oxygen is reduced by reactive oxygen species (ROS) which ultimately damage the cell [9-11]. This ROS gets destroyed by several enzymes such as mitochondrial and sarcoplasmic SODs (superoxide dismutase) [62]. ATP gets hydrolyzed in cardiomyocyte and is utilized for contractility apparatus (actomyosin) beside this it is also used to maintain ionic balance inside the cell [62]. Oxygen utilization is correlated with a concentration gradient, the electrochemical gradient across the mitochondrial membrane consists of a concentration gradient of 1PH unit (60mV) and an electric potential of 140mV (negative inside) [63]. In human cardiac tissue, ATP remains in equilibrium with creatine phosphate which acts as a temporary storage of ‘high energy’ phosphate bond with the utilization of the enzyme creatine kinase [63]. Normally the contractile mechanism of the cell is controlled by (Ca^++^) concentration of free cytoplasmic (Ca^++^) [1, 64]. Uniport mechanism is employed for the release of calcium from the sarcoplasmic reticulum as well as uptake from the outside cell *via* the sarcolemmal (Ca^++^) channel [65].

### 3.3 Mitochondrial Dysfunction and Cell Death

**Fig. (4):**
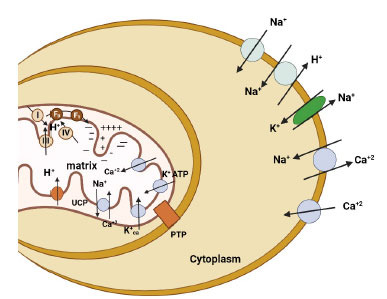
Cation transporter in heart cells.

Mitochondria are the primary sources of ATP production in myocytes beside the production of energy mitochondria also contribute to cell death in response to cellular stress such as overproduction of ROS, serum deprivation, and DNA damage [38]. Mitochondria are the organelles that undergo morphological changes through a process of fission and fusion [15]. In response to stress, mitochondria release some apoptotic factors such as cytochrome c, endonuclease G, and apoptosis-inducing factor (AIF) [66]. There are three types of cell death that occur in heart cells: necrosis, apoptosis, and autophagy. Autophagy is a response to the cellular phenomenon that degrades proteins and leads to organelles dysfunction [67]. Autophagy is characterized by the sequestration of cytoplasm in a double membrane vesicles called autophagosomes [68] (Fig. **4**).

**Fig. (5):**
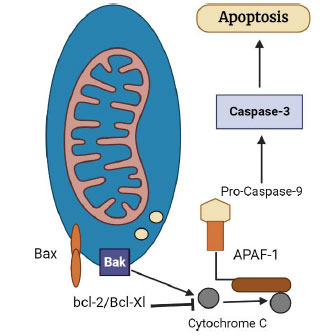
Mitochondrial dysfunction plays a critical role in cardiomyocyte apoptosis in the ischemic heart.

Necrosis is a passive form of cell death that is characterized by depletion of ATP as well as rapid disruption of the membrane which alters the integrity of the cells and results in the exocytosis of intracellular content to extracellular spaces. This released cellular material causes inflammation and damage to neighboring cells [53, 69]. In the apoptosis form of cell death, damaged cells are removed without causing inflammation. Apoptosis is also characterized by condensation of chromatin, DNA fragmentation, and cell shrinkage due to a reduction in cytoplasm and organelles [42, 53]. Apoptosis gets activated in cardiomyocytes by the induction of stressors such as increased ROS production, DNA damage, and cytokine production [2, 70]. The mitochondrial cell death pathway is regulated by pro and anti-apoptotic Bcl2 proteins. This protein shares four conserved Bcl2 homology (BH) domains. Anti-apoptotic Bcl-2 proteins, such as Bcl-2 and Bcl-XL, contain all four subtypes of BH domains (BH1-4) and promote cell survival (Fig. **5**) [71, 72].

## CONCLUSION

Mitochondria are crucial for sustaining life by continuous generation of energy which is essential for cell growth and development. Besides this, they also play a role in cell survival and cell death. In myocardial tissue, mitochondria occupy more than 25% of cytoplasmic space which ensures the continuous production of ATP by means of oxidative metabolism. The heart muscle uses this energy along with an electrochemical gradient for contractility purposes. During ischemia in the heart low perfusion of blood and oxygen supply to cardiac cells reduces, under this condition, anaerobic metabolism is activated within the mitochondria, thus accumulation of lactic acid occurs. Under this condition, TCA cycle also gets completely blocked and leads to the accumulation of NADH. At the stage of cellular stress likewise, overproduction of oxidative stress, ROS, cytokine activation, and calcium overload within the mitochondria membrane leads to dysfunction of mitochondria. Several patterns of cell death are being followed up likewise apoptosis and necrosis. In myocardial infarction, such prolonged ischemia leads to mitochondrial dysfunction and ultimately damage or even death of cardiac tissue.

## Figures and Tables

**Table 1:** Symptom differences between men and women in myocardial infarction [9].

**Symptoms More Frequent in Women**	**Studies (n of Total)**
Shortness of breath	6 of 10
Arm/shoulder pain	1 of 5
Abdominal pain	1 of 6
Back pain	5 of 6
Fatigue	2 of 3
Nausea	5 of 8
Dizziness	1 of 6
**Note: Symptoms more frequent in men**	
Chest pain	3 of 8
Chest pain as a primary symptom	1 of 1
Sweating	4 of 8
Belching	1 of 1
Hiccups	1 of 1

## LIST OF ABBREVIATIONS

AIF: Apoptosis-inducing Factor
MI: Myocardial Infarction
MPTP: Mitochondrial Permeability Transition Pore
IMM: Inner Mitochondrial Membrane
OMM: Outer Mitochondrial Membrane
AIF: Apoptosis Inducing Factor
BH: Bcl2 Homology
ROS: Reactive Oxygen Species
